# The group-based law enforcement mistrust scale: psychometric properties of an adapted scale and implications for public health and harm reduction research

**DOI:** 10.1186/s12954-022-00635-3

**Published:** 2022-06-03

**Authors:** Laura M. Johnson, Paul G. Devereux, Karla D. Wagner

**Affiliations:** School of Public Health, University of Nevada, Reno, 1664 N Virginia Street, Reno, NV 89557 USA

**Keywords:** Black/African American, Law enforcement, Risk environment

## Abstract

**Background:**

Negative encounters with law enforcement—direct and vicarious—fuel mistrust. When considered as part of the ‘risk environment’ in public health and harm reduction research, law enforcement mistrust may have broad implications. For example, fearing arrest may prevent someone from calling 911 when witnessing an overdose or lead to syringe-sharing and community spread of HIV. For people in the US who identify as Black or African American, these effects may compound, given the ways in which communities of color have been overpoliced. The purpose of this study is to investigate the psychometrics of an adapted scale of law enforcement mistrust—the Group-Based Law Enforcement Mistrust Scale (GBLEMS)—and evaluate its associations with racial and ethnic identity and experiences with law enforcement.

**Methods:**

This cross-sectional survey took place in a small city in the Western United States where only 3% of the population is Black or African American. The sample included Black or African American and Hispanic and Latina women at risk of HIV, and members of their social networks, yielding a diverse sample across racial, ethnic, and gender identities (*N* = 219). The GBLEMS is a 12-item scale adapted from the Group-Based Medical Mistrust Scale (GBMMS; Thompson et al. 2004). The current analysis evaluated the psychometric properties of the GBLEMS (reliability, exploratory factor analysis) and its associations with demographics, other race-based constructs, and experiences with law enforcement.

**Results:**

The GBLEMS demonstrated strong reliability (Cronbach’s alpha = 0.92) and exploratory factor analysis indicated that items loaded onto two factors—mistrust and disparities in treatment. There was also support for the scale’s construct validity. As hypothesized, GBLEMS scores were higher among respondents who identify as Black or African American, and among those who reported other experiences of racial discrimination, medical mistrust, and negative encounters with law enforcement.

**Conclusions:**

This study yielded support for the reliability and validity of the GBLEMS as a multi-item, two-factor scale measuring group-based law enforcement mistrust. When framing public health and harm reduction research in terms of the risk environment, law enforcement mistrust may be important to measure as part of a comprehensive approach that addresses persistent racial disparities.

## Background

The ‘risk environment’ [1] provides a framework for understanding how broader contextual forces, including interactions with law enforcement and the criminal justice system, shape individual-level health outcomes. At the individual-level, encounters with law enforcement can serve as an impediment to accessing HIV prevention services or engaging with emergency medical services during an acute health crisis [2, 3] because calling 911 is often seen as equivalent to calling the police [4]. For example, Rhodes [1] described a case in which stricter policing coincided with higher levels of syringe-sharing among people who inject drugs (PWIDs) in Kathmandu. Other studies among PWIDs in the US have found associations between actual or feared encounters with the police and syringe-sharing and use of syringe services programs [5–7]. And in the case of opioid overdoses, even when policy measures are in place (such as Good Samaritan laws) to provide protections against some criminal justice consequences, individuals still fear those consequences [8] and cite their own previous negative experiences as the reason why they are reluctant to call 911 [9]. At the structural level, individuals who lost their social security benefits due to policies that criminalize the possession of syringes were more likely to participate in illegal activities, share syringes, and inject more frequently than individuals who retained their social security benefits [6]. In other words, there are identified pathways between the policies and practices of policing and individual-level and community-level outcomes.

Compared to their White counterparts, people living in the US who identify as Black or African American are more likely to report negative encounters with law enforcement and associated mistrust. People who identify as Black or African American are also overrepresented among those living with HIV [10], and experience worse health [11, 12] and criminal justice [13–16] outcomes compared to their White counterparts. Calls have been made to harmonize public health and law enforcement priorities to address risk factors for HIV [17, 18] and prevent opioid overdose deaths [19]. Nonetheless, fear of or mistrust in law enforcement is still a significant driver of public health outcomes, particularly among racial and ethnic minorities in the US. To that end, and in a large national sample of PWIDs, Cooper et al. [20] found that HIV-related risk environments in the US are racialized, such that Black or African American PWIDs were more likely to live in areas with higher poverty, higher crime rates, and worse access to drug treatment; and less likely to live in areas where laws facilitate sterile syringe access. Considering policing policy and practices into this racialized risk environment may help to further contextualize some of these disparities.

Since its inception in 2013, the Black Lives Matter social movement has increased the visibility of and fostered a national discourse about law enforcement’s differential treatment of minoritized people in the US. Black or African American and White people diverge substantially on their views toward police, with 56% of White adults saying they have a great deal/quite a lot of confidence in the police, compared to only 19% of people who are Black or African American [21]. However, both a majority of people who are Black or African American (84%) and White (63%) agree that African Americans are treated less fairly by the police [22]. Not merely a policing issue, however, Black or African American men’s reports of police discrimination (e.g., accusations of selling drugs, getting pulled over, being verbally and physically abused) are associated with other experiences of racial discrimination and depression [23]. Other studies have begun to explore the relationship between experiences with police brutality and medical mistrust, finding that reporting negative encounters with law enforcement was associated with higher levels of medical mistrust [24] and greater odds of having unmet medical care needs [25].

Considering racial disparities within law enforcement and public health contexts is complex. In addition to racial tensions between police and people who are Black or African American, police themselves face tensions within expanding and conflicting aspects of their role [26]. While they have always been enforcers of the law, more recently they have been charged with implementing measures that emphasize public health and harm reduction values [27]. Recent policy efforts attempt to expand the focus of law enforcement from a “crime-control to addiction-treatment” [28] model of policing, and these shifting and competing priorities have caused role strain and frustration among law enforcement officers [28]. While the purpose of these policies was to decriminalize certain aspects of drug possession and use, a qualitative study in California found that law enforcement officers employ adaptive techniques to work around these policies, such as charging PWIDs for associated crimes (e.g., possession for sales or transport) as opposed to more minor drug-related crimes (e.g., possession) so that these decriminalizing/diversion policies no longer apply [28]. The introduction of harm reduction-oriented policies may be creating a fundamental mismatch within law enforcement values, and until these tensions are resolved, officers’ active circumvention of these policies could further undermine individual and public trust in law enforcement.

While there have been some attempts to measure mistrust in police and how it varies by race as mentioned above, these works have limitations. For example, Sharp and Johnson [29] found that trust in police was lower for people who are Black or African American compared to people who are White, and that various individual-level (e.g., demographics, racial group identification) and community-level (e.g., Black representation on the police force, crime indices) factors helped explain some of these racial differences in mistrust. However, this study employed a single-item measure of mistrust, which fails to take into account the multidimensional nature of the concept [29], measuring suspicion of actors/systems but not perceptions of differential treatment across racial groups [30]. Another study revealed that these measurement choices (single vs. multi-item questionnaire) had implications for whether mistrust was detected. In a sample of first- and second-generation immigrants, a single-item measure of mistrust failed to detect differences between immigrant groups while a multi-item measure did [31]. The authors conclude that their findings underscore the need to consider the multidimensionality of the concept itself; this study does just that.

The current study extends existing work with a novel adaptation of the previously validated Group Based Medical Mistrust Scale (GBMMS; [30, 32]), adapted for a broader study examining social network and structural determinants of HIV testing among Black or African American and Hispanic and Latina women and members of their social networks. The new “group based” scale not only captures mistrust in law enforcement, but also anchors these perceptions in relation to one’s group membership, which “assesses the tendency to distrust those who do not belong to one’s ethnic group and/or distrust systems that do not belong to one’s ethnic group based on a legacy of racism and unfair treatment” [30]. In the current analysis, we describe and provide psychometric evidence for the validity of this adapted scale—the Group-Based Law Enforcement Mistrust Scale (GBLEMS)—and explore its associations with racial and ethnic identity and experiences with law-enforcement. We hypothesize that (1) Black or African American respondents will report greater mistrust than other racial groups, and (2) greater levels of law enforcement mistrust will be associated with greater levels of other race-based measures of discrimination, medical mistrust, and negative experiences with law enforcement.

## Methods

### Setting and sample

The study is based on survey data collected as part of a broader project exploring HIV risk and HIV testing in a small city in the US West. While the primary focus was on Black or African American and Hispanic and Latina women at-risk for HIV, the study also employed a one-step referral design such that respondents could refer members of their networks to participate, which resulted in a more diverse sample by gender, race, and ethnicity. When reporting on race and ethnicity in this manuscript, we referred to the guidelines presented in Flanagin et al. [33]. While many participants in this study identify as Black or African American (43%), Hispanic or Latino (17%), or more than one race or ethnicity (23%), the city’s population is much different, with only 3% identifying as Black or African American, 25% as Hispanic or Latino, and 3% as multiracial. Similarly, racial and ethnic minorities are underrepresented in the local police force, where less than 2% identify as Black or African American and 11% identify as Hispanic or Latino. Further, the metropolitan area had the 11^th^ highest rate of police killings in the United States between 2013 and 2020 [34]. In short, this is a regional context where (1) people of color are in the minority (both absolutely and relatively), (2) their interactions with law enforcement officers will often be with members of a majority racial or ethnic group, and (3) per capita, police killings are the highest in the state and among the highest in the nation. Data were collected between 2016 and 2018 through interviewer-administered surveys that often lasted between one and two hours.

Participants were recruited through convenience sampling techniques across the community. Though recruitment mode is not central to this analysis, it is important to note that recruitment occurred in two stages. First, index participants were recruited based on the following criteria: adult women (cisgender or transgender), self-identified as Black or African American or Hispanic or Latina, willing to refer others, negative/unknown HIV status but at risk for HIV as determined by screening questions. Upon completing the survey, participants were provided coupons to recruit members of their social networks to participate in the study. The inclusion criterion for referred participants was only that they be connected to an index participant (by presenting a coupon or providing the index participant’s name). Referred participants were not asked to recruit further. The survey covered topics such as self-reported HIV risk behaviors and testing, knowledge and attitudes around HIV, other social-psychological constructs (such as law enforcement mistrust and medical mistrust, among others), and aspects of their social networks (which are not included in the current analysis). This study was reviewed by a Community Advisory Board, which included people of color and local advocates and community leaders, and the study was approved by the Institutional Review Board at the University of Nevada, Reno. Participants provided written consent and were compensated $40 for their participation in the study and index participants were also eligible to receive $5 per referred participant they recruited into the study (up to 5 referrals) Table 1.

**Table 1:** Demographic Characteristics of the Sample (*N* = 219)

Demographic variables and other psycho-social constructs	*N* (%) or Mean (SD)
Sex/Gender	
Male	54 (25.7%)
Female	161 (73.5%)
Transgender female	4 (1.8%)
Race/Ethnicity	
Black/African American	94 (42.9%)
Hispanic or Latino	28 (12.8%)
White	37 (16.9%)
Other	10 (4.6%)
Multiracial	50 (22.8%)
Any Black	131 (59.8%)
Homeless in past 6 months	110 (50.2%)
Age	37.2 (13.1)
Education	12.9 (2.1)
Monthly income	997.3 (892.4)
How many times in the past 6 months have you felt unsafe in your neighborhood?	
None	98 (44.7%)
Once	21 (9.6%)
Twice	24 (11.0%)
3–10 times	35 (16.0%)
> 10 times	41 (18.7%)
Multiple discrimination scale race subscale (MDS-R)	2.7 (2.4)
Group-based medical mistrust scale (GBMMS)	2.6 (0.8)
*Illicit activities and experiences with law enforcement*	
Injection drug use (past 6 m)	41 (18.7%)
Receptive syringe and paraphernalia sharing (past 6 m; *missing n* = *3*)	20 (9.3%)
Sex with recently incarcerated partner (past 6 m; *missing n* = *13*)	40 (19.4%)
Ever ticketed (excluding parking)	150 (68.5%)
Ever arrested	163 (74.4%)
Ever spent night in jail	52 (23.7%)
Stopped by law enforcement because of your race (past 12 m)	46 (21.0%)
Arrested because of your race (past 12 m; *missing n* = *1*)	19 (8.7%)
Treated poorly or made to feel inferior by law enforcement because of your race? (past 12 m; *missing n* = *2*)	58 (26.7%)
Have law enforcement officials ever informed you about social services, drug treatment, needle exchange programs or other services available to you?	64 (29.2%)

### Measures

The outcome of interest is the GBLEMS. Items were drawn from the GBMMS [30]. All items were retained but modified (through word/phrase substitution) for a law enforcement context. For example, references to ‘doctors and healthcare workers’ were replaced with ‘law enforcement officers,’ language about ‘modern medicine’ was replaced with ‘the criminal justice system,’ and so on. Specifically, item #9 from the medical mistrust scale was worded as “Doctors and health care workers do not take the medical complaints of people of your racial group seriously” but in the current scale, it was modified to be “Law enforcement officers do not take the complaints of people of your racial group seriously.” For each item, response options were: 1 = Strongly Disagree, 2 = Disagree, 3 = Neither Agree nor Disagree, 4 = Agree, and 5 = Strongly Agree, just as in the GBMMS. A complete list of scale items is presented in Table 2.

**Table 2:** Descriptive statistics of the group-based law enforcement mistrust scale (GBLEMS; *N* = 218)

	Mean (SD)	% Disagree	% Neutral	% Agree
Overall score	3.36 (0.87)	–	–	–
*Individual items*				
1. Law enforcement officers sometimes hide information from people who belong to your racial group	3.63 (1.26)	21%	17%	61%
*2. Law enforcement officers have the best interests of people of your racial group in mind	2.51 (1.10)	54%	26%	20%
3. People of your racial group should not confide in law enforcement officers because it will be used against them	3.23 (1.19)	31%	27%	42%
4. People of your racial group should be suspicious of information from law enforcement officers	3.18 (1.16)	33%	24%	43%
5. People of your racial group cannot trust law enforcement officers	3.12 (1.14)	33%	27%	40%
6. People of your racial group should be suspicious of the criminal justice system	3.53 (1.21)	25%	15%	60%
7. Law enforcement officers treat people of your racial group like “animals.”	3.21 (1.19)	30%	25%	45%
*8. People of your racial group receive the same protection from law enforcement officers as people from other groups	2.58 (1.20)	56%	14%	30%
9. Law enforcement officers do not take the complaints of people of your racial group seriously	3.27 (1.12)	30%	22%	48%
*10. People of your racial group are treated the same as people of other groups by law enforcement officers	2.36 (1.13)	64%	15%	21%
*11. In most courts, people of different racial groups receive the same kind of treatment from the judge	2.60 (1.20)	54%	17%	29%
12. You have personally been treated poorly or unfairly by law enforcement officers because of your race	3.17 (1.30)	42%	12%	46%

^*^Reverse-coded for scale creation

Demographic characteristics were all self-reported and included sex and gender (female, male, transfemale, transmale, gender queer/nonbinary, other), race and ethnicity (Black or African-American, African, Afro-Caribbean, Black Other, Hispanic or Latino, Asian, American Indian/Alaskan Native, Native Hawaiian/Pacific Islander, White; select all that apply), age and education (both in years), and income (monthly dollar amount). A binary measure assessed past six-month homelessness (1 = Yes). An ordinal measure assessed how many times the respondent felt unsafe in their neighborhood (0 = None, 1 = Once, 2 = Twice, 3 = Three to ten times, 4 = More than ten times; [35]). Two other constructs included experiences of discrimination and group-based medical mistrust. Experiences of racial discrimination were measured with the Multiple Discrimination Scale (Race Subscale; [36]), which is a series of ten items and respondents indicate if they have experienced that form of discrimination in the past year. Two sample items are: “Were you denied a job or did you lose a job because of your race?” and “Were you physically assaulted or beaten up because of your race?” Race-based medical mistrust was measured with the GBMMS [30], which consists of twelve items averaged to create a mean score. Two sample items are, “Doctors and health care workers sometimes hide information from patients who belong to your racial group.” and “People of your racial group cannot trust doctors and health care workers.”

Experiences with law enforcement were measured with several binary items assessing indirect and direct exposures. One question asked if the respondent had sex with someone who was recently incarcerated (1 = Yes), which represents an indirect/vicarious experience. Direct experiences included ever having been ticketed (excluding parking tickets), been arrested, or spent the night in jail (asked separately, 1 = Yes). One question asked participants whether law enforcement had ever informed them about social services, drug treatment, syringe exchange programs, or other services (1 = Yes). Given that they could prompt interactions with law enforcement, measures of two illicit activities were included: past 6-month injection drug use and past 6 month receptive syringe and paraphernalia sharing (asked separately, 1 = Yes). Finally, three race-based questions asked if respondents had ever been stopped by law enforcement, arrested, or treated poorly because of their race.

### Analytic strategy

Prior to analysis, several variables were recoded. Due to small sample size and under the guidance of the Community Advisory Board, respondents who selected Black or African-American, African, Afro-Caribbean, Black Other were combined to indicate Black or African American racial identity. Recoded racial and ethnic categories included Black or African American (only Black or African American selected), Latino or Hispanic (with or without White selected), Non-Hispanic White, Other (which combined Asian, American Indian/Alaskan Native, Native Hawaiian/Pacific Islander due to small sample sizes), and Multiracial (more than one race or ethnicity selected). Black or African American racial identity was also considered dichotomously for some analyses and ‘Any Black or African American’ was recoded to include those who identify as Black or African American as well as those who are multiracial and selected Black or African American with another racial or ethnic identity. The GBLEMS was created by reverse coding four items and then taking the average score across all twelve items, where higher values indicate more mistrust. Taking the average as opposed to the sum allowed us to retain three cases where respondents skipped a single item. One individual skipped 11 out of 12 items and was excluded from the GBLEMS analysis. In one instance, individual scale items were analyzed as categorical variables: ‘Disagree’ (including ‘Strongly Disagree’ and ‘Disagree’), ‘Neutral’ (including ‘Neither Agree nor Disagree’), and ‘Agree’ (including ‘Strongly Agree’ and ‘Agree’).

Analysis took place in four steps. First, we described the characteristics of the sample. Second, we described the overall scale and individual item scores of the GBLEMS and explored the psychometric properties of the scale (Cronbach’s alpha and an exploratory factor analysis). An exploratory factor analysis was conducted using the SAS PROC FACTOR procedure. Extracted factors were then rotated using an oblique technique (allowing for correlated factors) via the promax option in SAS. Third, we compared GBLEMS (individual items and the overall scale and subscales) by race or ethnicity; between group *t*-tests were used to detect statistical significance between subgroups. Finally, to assess the validity of this adapted scale, the GBLEMS was correlated with other constructs—for all respondents together and then among Black or African American and White participants separately—such as demographics, medical mistrust, experiences with discrimination, and experiences with law enforcement. Missing data were handled through pairwise deletion.

## Results

### Sample characteristics

A description of the sample (*N* = 219) is presented in Table 1. By design, the sample is weighted more heavily toward women and people who are Black or African American. The sample was 74% cisgender women (*n* = 161), 2% transgender women (*n* = 4), and 26% cisgender men (*n* = 54). A plurality of respondents (43%, *n* = 94) were Black or African American, followed by multiracial (23%, *n* = 50), White (17%, *n* = 37), Hispanic or Latino (13%, *n* = 28), and Other (5%, *n* = 10). Sixty percent of respondents (*n* = 131) indicated any Black or African American racial identity when race was recoded to include those who are multiracial. Half the sample (50%, *n* = 110) had been homeless in the past six months. The average respondent was 37 years old with 13 years of education and a monthly income just under one thousand dollars ($997). Approximately 45% of respondents (*n* = 98) said they had never felt unsafe in their neighborhood in the past six months and 55% (*n* = 121) had felt unsafe at least one time, including 19% (*n* = 41) who felt unsafe more than ten times in the past six months. The average score on the discrimination scale was 2.7 (SD = 2.4) out of ten listed examples of racial discrimination. The average score on the GBMMS was 2.6 (out of 5; SD = 0.8), which, for reference, is between ‘disagree’ and ‘neither agree nor disagree’ on the five-point scale. With regard to illicit activities, nineteen percent of respondents (*n* = 41) had recently injected drugs and 9% (*n* = 20) had shared syringes or other paraphernalia. For indirect experiences with law enforcement, nearly one-in-five (19%, *n* = 40) respondents had a past 6-month sex partner who had recently been incarcerated. And concerning their own lifetime experiences with law enforcement, 69% (*n* = 150) had been ticketed, 74% (*n* = 163) had been arrested, and 24% (*n* = 52) had spent the night in jail. Regarding experiences with law enforcement in the past year, 21% of respondents (*n* = 46) believed they had been stopped by police because of their race, 9% (*n* = 19) believe they were arrested because of their race, and 27% (*n* = 58) believe they were treated poorly because of their race. Nearly one-third of respondents (29%, *n* = 64) said that law enforcement had informed them about available social services such as drug treatment or syringe exchange programs.

### Description and validation of the group-based law enforcement mistrust scale (GBLEMS)

A descriptive analysis of the GBLEMS is presented in Table 2, organized as a summary of the overall scale (mean, standard deviation) and a breakdown by each individual item (mean, standard deviation, and percent disagree/agree). The average score on this 12-item scale was 3.36 (SD = 0.87), which is between ‘neither agree nor disagree’ and ‘agree’ of the five-point response options. More than half of respondents indicated mistrust for the following six items:

- “People of your racial group are treated the same as people of other groups by law enforcement officers” (64% disagree; n = 140)
- “Law enforcement officers sometimes hide information from people who belong to your racial group” (61% agree; *n* = 134)
- “People of your racial group should be suspicious of the criminal justice system (60% agree; *n* = 131),
- “People of your racial group receive the same protection from law enforcement officers as people from other groups” (56% disagree; *n* = 121)
- “Law enforcement officers have the best interests of people of your racial group in mind” (54% disagree; *n* = 117), and
- “In most courts, people of different racial groups receive the same kind of treatment from the judge.” (54% disagree; *n* = 117).

At least 40% of respondents indicated mistrust for the remaining six items.

The overall scale had strong internal consistency with a standardized Cronbach’s alpha of 0.92. With regard to the factor analysis, and by examining the scree plot and eigenvalues > 1, it appears there are two factors to the scale (see Table 3). Nine of the 12 items loaded on Factor 1 and three items loaded on Factor 2 (indicated in bold in the corresponding columns). Interpreting the two factors, Factor 1 is about mistrust of law enforcement generally, whereas Factor 2 is about disparities in treatment by law enforcement (Cronbach’s alpha for Factor 1 = 0.90; Factor 2 = 0.84).

**Table 3:** Group Based Law Enforcement Mistrust Scale (GBLEMS): Results from an Exploratory Factor Analysis^◊^

	Mistrust Subscale Factor 1	Disparities in Treatment Subscale Factor 2
Eigenvalue	17.22	2.21
Proportion of Variance	89%	11%
Individual Items^		
4. People of your racial group should be suspicious of information from law enforcement officers	**.873**	-.059
5. People of your racial group cannot trust law enforcement officers	**.711**	.107
7. Law enforcement officers treat people of your racial group like “animals.”	**.610**	.205
9. Law enforcement officers do not take the complaints of people of your racial group seriously	**.605**	.197
3. People of your racial group should not confide in law enforcement officers because it will be used against them	**.599**	.104
6. People of your racial group should be suspicious of the criminal justice system	**.587**	.203
1. Law enforcement officers sometimes hide information from people who belong to your racial group	**.465**	.260
*2. Law enforcement officers have the best interests of people of your racial group in mind	**.429**	.368
12. You have personally been treated poorly or unfairly by law enforcement officers because of your race	**.419**	.119
*10. People of your racial group are treated the same as people of other groups by law enforcement officers	-.012	**.934**
*8. People of your racial group receive the same protection from law enforcement officers as people from other groups	.173	**.654**
*11. In most courts, people of different racial groups receive the same kind of treatment from the judge	.133	**.605**

^Factor pattern loadings are** bolded** and sorted in descending order

^◊^Rotated factor loadings for 2-factor model

^*^Reverse-coded

Table 4 displays means and standard deviations of GBLEMS (overall scale, subscales, and individual items) by racial and ethnic identity. With regard to the overall score, Black or African American respondents (mean = 3.64) reported significantly more mistrust than people who were Hispanic or Latino (mean = 3.00), White (mean = 2.97), or some other race (mean = 2.71; all *p’s* < 0.05). People who identify as multiracial were also more likely to report law enforcement mistrust (mean = 3.44) when compared to people who are White, Hispanic or Latino, or some other race (*p’s* < 0.05). Looking at the individual items, Black or African American respondents reported statistically significantly more group-based law enforcement mistrust compared to at least one other racial and ethnic group across all twelve items. These results lend support to Hypothesis 1 that identifying as Black or African American is associated with reporting greater group-based law enforcement mistrust.

**Table 4:** Descriptive statistics of the GBLEMS and its association^ with race or ethnicity^○^ (*N* = 218)

	Overall *N* = 218	Black/African American^1^ *N* = 94	Hispanic or Latino^2^ *N* = 28	White^3^ *N* = 37	Other^4^ *N* = 10	Multiracial^5^ *N* = 49
	Mean (SD)	Mean (SD)	Mean (SD)	Mean (SD)	Mean (SD)	Mean (SD)
Overall Scale	3.36 (0.87)	3.64^2,3,4^ (0.83)	3.00 (0.71)	2.97 (0.78)	2.71 (0.71)	3.44^2,3,4^ (0.89)
Mistrust subscale	3.31 (0.88)	3.60 (0.83)^2,3,4^	2.96 (0.76)	2.88 (0.82)	2.69 (0.81)	3.41 (0.89)^2,3,5^
Disparities in treatment subscale	3.49 (1.03)	3.74 (1.01)^2,3,4^	3.10 (0.87)	3.24 (0.96)	2.77 (0.93)	3.54 (1.07)^5^
*Individual items*						
1. Law enforcement officers sometimes hide information from people who belong to your racial group	3.63 (1.26)	3.89^3,4^ (1.14)	3.86^3,4^ (0.97)	2.97 (1.30)	2.80 (1.48)	3.68^3^ (1.33)
*2. Law enforcement officers have the best interests of people of your racial group in mind	2.51 (1.10)	2.18 (1.00)	2.96^1^ (1.07)	2.89^1^ (1.07)	2.78 (1.20)	2.54 (1.11)
3. People of your racial group should not confide in law enforcement officers because it will be used against them	3.23 (1.19)	3.48^3,4^ (1.20)	3.00 (1.05)	3.00 (1.18)	2.60 (1.17)	3.18 (1.18)
4. People of your racial group should be suspicious of information from law enforcement officers	3.18 (1.16)	3.52^2,3,4^ (1.04)	2.79 (1.07)	2.76 (1.19)	2.50 (1.18)	3.20 (1.19)
5. People of your racial group cannot trust law enforcement officers	3.12 (1.14)	3.43^2,3^ (1.07)	2.71 (1.18)	2.68 (1.13)	2.90 (0.88)	3.14 (1.15)
6. People of your racial group should be suspicious of the criminal justice system	3.53 (1.21)	3.78^2,3,4^ (1.11)	3.21 (1.23)	2.95 (1.29)	3.00 (0.94)	3.78^3^ (1.21)
7. Law enforcement officers treat people of your racial group like “animals.”	3.21 (1.19)	3.53^2,3,4^ (1.04)	2.67 (1.18)	2.70 (1.13)	2.40 (1.07)	3.43^2,3,4^ (1.24)
*8. People of your racial group receive the same protection from law enforcement officers as people from other groups	2.58 (1.20)	2.21 (1.14)	3.11^1^ (1.07)	2.95^1^ (1.15)	3.20^1^ (0.79)	2.57 (1.27)
9. Law enforcement officers do not take the complaints of people of your racial group seriously	3.27 (1.12)	3.59^2,3^ (1.04)	2.82 (1.06)	2.73 (1.04)	2.90 (1.10)	3.39^2,3^ (1.13)
*10. People of your racial group are treated the same as people of other groups by law enforcement officers	2.36 (1.13)	2.13 (1.06)	2.71^1^ (1.18)	2.65^1^ (1.09)	3.10^1,5^ (1.20)	2.24 (1.13)
*11. In most courts, people of different racial groups receive the same kind of treatment from the judge	2.60 (1.20)	2.43 (1.20)	2.89 (1.17)	2.68 (1.11)	3.40^1^ (1.17)	2.55 (1.26)
12. You have personally been treated poorly or unfairly by law enforcement officers because of your race	3.17 (1.30)	3.38^2,4^ (1.23)	2.57 (1.17)	3.03^4^ (1.21)	2.00 (1.05)	3.45^2,4^ (1.39)

^*^Reverse-coded for scale creation

^^^Significant differences (between group *t*-test; p < .05) between racial and ethnic subgroups indicated with superscripts Black^1^, Hispanic or Latino^2^, White^3^, Other^4^, and Multiracial^5^ with the superscript notation is placed at the higher value

^○^Race or ethnicity defined as: Black/African American = Black; Hispanic or Latino = with or without White; White = Non-Hispanic White; Other = Asian, American Indian/Alaskan Native, Native Hawaiian/Pacific Islander; Multiracial = More than one race or ethnicity selected

### GBLEMS’ associations with demographics and illicit activities and experiences with law enforcement

Table 5 displays the association between the GBLEMS and other characteristics related to demographics, illicit activities, and experiences with law enforcement. Indicating any Black or African American racial identity was associated with law enforcement mistrust as measured by the GBLEMS (Pearson *r* = 0.31, *p* < 0.0001), as was education (Pearson *r* = 0.14, *p* < 0.05), such that people who identify as Black or African American and people with more education scored higher on the GBLEMS. In the racial subgroup analysis, age was negatively associated with the GBLEMS (Pearson *r* = − 0.21, *p* < 0.05). In other words, among participants who identified as Black or African American, law enforcement mistrust decreased as age increased. Among White respondents, those who had experienced recent homelessness scored higher on the GBLEMS (Pearson *r* = 0.47, *p* < 0.05*)*. For all participants, as reports of race-based discrimination increased, so did law enforcement mistrust (Pearson *r* = 0.32, *p* < 0.0001). A similar positive association was found for medical mistrust (GBMMS) and the GBLEMS (Pearson *r* = 0.50, *p* < 0.0001) where greater *medical* mistrust was associated with greater *law enforcement* mistrust. In the subgroup analysis, having experienced discrimination (MDSR) was associated with law enforcement mistrust among Black respondents only (Pearson *r* = 0.37, *p* < 0.05) and medical mistrust remained significant for both groups (Black or African American Pearson *r* = 0.51, *p* < 0.0001; White Pearson *r* = 0.53, p < 0.01). In other words, the more someone perceived themselves experiencing race-based discrimination (for Black and African American respondents) and the more someone expressed medical mistrust (all respondents), the more likely they were to express race-based law enforcement mistrust. Examining self-reported illicit activities and experiences with law enforcement, four items were positively associated with GBLEMS for all respondents and among Black respondents: having a past 6-month sexual partner who had been recently incarcerated (All: Pearson *r* = 0.22, *p* < 0.01; Black or African American Pearson *r* = 0.28, *p* < 0.01), having been stopped by law enforcement because of your race (All: Pearson *r* = 0.32, *p* < 0.0001; Black or African American Pearson *r* = 0.40, *p* < 0.0001), having been arrested because of your race (All Pearson *r* = 0.19, *p* < 0.01; Black or African American Pearson *r* = 0.24, *p* < 0.05), and having been treated poorly or made to feel inferior by law enforcement because of your race (All: Pearson *r* = 0.37, *p* < 0.0001; Black or African American Pearson *r* = 0.32, *p* < 0.01). Two non-race-based variables were significant among White respondents: past 6-month syringe/paraphernalia sharing (Pearson *r* = 0.44, *p* < 0.01), and having ever been arrested (Pearson *r* = 0.35, *p* < 0.05). These results support Hypothesis 2 that law enforcement mistrust would be greater among respondents who also report other race-based measures of discrimination, medical mistrust, and negative experiences with law enforcement.

**Table 5:** GBLEMS correlations among all participants and comparing Black or African American and White participants

	All *N* = 218				Black or African American *N* = 94				White *N* = 37		
	Pearson correlation	*p*-value			Pearson correlation	*p*-value			Pearson correlation	*p*-value	
*Demographic variables and other psycho-social constructs*											
Any Black or African American	**.31**	**< .0001**	***		**–**	**–**			**–**	**–**	
Homeless in past 6 months	.11	.11			.01	.95			**.47**	**< .01**	**
Age	− .04	.53			− **.21**	**< .05**	*		.04	.79	
Education	**.14**	**.03**	*		.16	.12			.02	.90	
Monthly income	− .04	.60			− .10	.32			.08	.64	
Feeling unsafe in neighborhood	.11	.09			.05	.60			.12	.47	
Multiple discrimination scale (race subscale: MDSR)	**.32**	**< .0001**	***		.37	**< .01**	**		.21	.20	
Group-based medical mistrust scale (GBMMS)	**.50**	**< .0001**	***		**.51**	**< .0001**	***		**.53**	**< .01**	**
*Illicit activities and experiences with law enforcement*											
Injection drug use (past 6 m)	-.03	.61			.02	.85			.31	.06	
Receptive syringe and paraphernalia sharing (past 6 m)	.04	.53			.03	.81			**.44**	** < .01**	**
Sex with recently incarcerated partner (past 6 m)	**.22**	** < .01**	**		.**28**	** < .01**	**		.28	.10	
Ever ticketed (excluding parking)	.04	.52			.04	.67			.07	.68	
Ever arrested	.10	.12			.11	.30			**.35**	**.03**	*
Ever spent night in jail	.12	.08			.10	.33			.32	> .05	
Have law enforcement officials ever informed you about social services, drug treatment, needle exchange programs or other services available to you?	.04	.56			.07	.51			.08	.66	
Stopped by law enforcement because of your race (past 12 m)	**.32**	** < .0001**	***		**.40**	** < .0001**	***		.16	.15	
Arrested because of your race (past 12 m)	**.19**	** < .01**	**		**.24**	**.02**	*		.15	.37	
Treated poorly or made to feel inferior by law enforcement because of your race? (past 12 m)	**.37**	** < .0001**	***		**.32**	** < .01**	**		.26	.12	

Bold terms indicate statistically significant correlations

^*^p < .05, **p < .01, ***p < .0001

## Discussion

The results of this study indicate strong internal consistency and good construct validity for the GBLEMS as a multi-item, multi-dimensional measurement tool for race-based law enforcement mistrust. In particular, the items on this scale hung together as a cohesive construct and loaded onto two factors (of which, the items in Factor 2 were identical to the scale on which this work was based [30]). An important next step in this validation process would be to explore the GBLEMS in a larger, population-based sample across other settings and with more advanced modeling techniques that could account for the complex ways in which these variables are associated with law enforcement mistrust. These future efforts would strengthen the validity and generalizability of our findings. There are two key takeaways from the current analysis. First, this scale supports the idea that Black or African American individuals see themselves as receiving differential and targeted treatment by law enforcement in relation to their own racial identity. These findings align with the growing body of scientific evidence supporting this perspective as well as the collective sentiments expressed through the Black Lives Matter social movement. Second, the study explores the association of the scale with direct experiences that may engender mistrust such as being stopped, arrested, and treated poorly by law enforcement because of one’s race. Findings suggest that the more negative interactions one has with the police, the more likely an individual is to have mistrust in law enforcement. Further, the findings from this study are useful, considering that law enforcement officers often interface with people who are similar to our sample: people who are homeless (who may need to be connected to social services), with people who engage in sex work (who may be at risk of HIV), and people who inject drugs (who may be at risk of overdose). Therefore, efforts to synthesize public health initiatives into existing policing practices should consider perceptions of police among these groups. These takeaways should be considered in light of efforts to integrate public health and policing, especially considering the ways in which minoritized communities have been policed and how this may undermine current efforts.

Future directions for research should be contextualized in relation to the current study’s limitations. First, the one-step referral design allowed for individuals who identify as White to participate, as long as they were referred by a respondent who was Hispanic or Latino or Black or African American. As a result, White participants in our sample may be different from other White participants in the region who may not have a racially or ethnically diverse social network. Specifically, White participants in our sample may be more attuned to racial injustices experienced by racialized people than the average White person. Even still, this scale was able to discern racial differences in law enforcement mistrust, which we identify as a strength. As a result, our findings about racial differences may be understated compared to what may be found in a more representative sample. Second, our sample was drawn from a population where people who identify as Black or African American are underrepresented as compared to other areas of the US. Given the extent of this underrepresentation, our Black and African American respondents may be targeted by police more than a counterpart in a more diverse community or may otherwise have different experiences than people living in more diverse communities. Third, while the current scale was adapted from the Group-Based Medical Mistrust Scale (GBMMS [30]) the current law enforcement mistrust scale (GBLEMS) did not retain a similar 3-subscale structure that was present in the original work and only two factors emerged in the present analysis. It may be the case that the dimensionality of law enforcement mistrust is not the same as medical mistrust or that the items failed to capture the full scope of the multidimensionality. Therefore, it is recommended that the scale’s factor structure be examined using larger and other diverse samples. The GBLEMS was created using word/phrase substitution to translate the GBMMS from a healthcare context to a law enforcement context and future research should explore a full range of theoretically relevant constructs around group-based law enforcement mistrust. However, our results provide evidence that in its current form, this scale does capture this concept broadly with strong indicators for validity and reliability. Finally, the current analysis was not the primary focus of the broader project and cross-sectional surveys of the sort employed in the parent study are—by design—unable to address causal pathways. Our study was, however, able to establish some associations that would be worthy of extension and application in the context of a risk environment framework. With the validation of the scale as the primary focus of this study, future studies could employ the GBLEMS in a study design that is better suited for exploring the causal link between law enforcement mistrust and outcomes such as HIV risk or calling 911 in response to an opioid overdose.

Employing a risk environment framework [1] provides further impetus to consider how policing policies and strategies have implications for broader public health concerns. For example, policies that divert PWIDs toward harm reduction resources (i.e., syringe service programs) and away from criminal justice pipelines (e.g., drug courts, law enforcement-assisted diversion) may lower the burden on the criminal justice system while improving individual HIV risk and reducing community-level spread of HIV. Some of these strategies may already be integrated into local policing procedures as 29% of respondents in the current study indicated they had been informed about various social services, drug treatment and syringe exchange programs by law enforcement. However, while increasing the *availability* of services and *informing* individuals about them is important, mistrust may still serve as a barrier to *accessing* such services. A policy change could improve one aspect of the risk environment, but its impact may not become fully realized if other aspects of the risk environment, such as perceptions of law enforcement, do not also shift. For example, a policy may require law enforcement officers to carry and administer naloxone to reverse opioid overdoses, but the impact may be muffled if people mistrust law enforcement so much that they do not call 911. To be sure, this mistrust may be justifiable as some jurisdictions with Good Samaritan laws allow for the prosecution of ‘drug induced homicides’ as a workaround. As a result, some scholars have argued that this workaround could have a chilling effect on bystanders’ willingness to report an overdose [37]. Having the ability to measure law enforcement mistrust can help communities understand if enacted policy changes that embrace harm reduction strategies have any meaningful impact on perceptions. Additionally, minoritized communities are overpoliced [13, 14, 16] and considering harm reduction alongside policing strategies presents an opportunity to close existing and persistent equity gaps. Having a race-based measure of law enforcement mistrust can highlight how perceptions vary across racial subgroups, which may be especially useful in meeting people where they’re at, a central tenet of harm reduction. Finally, and as mentioned above, having harm reduction policies in place does not guarantee that police won’t find workarounds and other ways to criminalize PWIDs [28]. As a result, indicators of race-based law enforcement mistrust may remain unchanged, or people may become more mistrustful because they remain unfairly targeted by policing strategies. By considering broader factors such as mistrust in law enforcement, public health interventions can expand their focus from an individualistic orientation to a community-level, collective orientation [1]. This shift combats a victim-blaming narrative and compels public health practitioners to consider the role of non-health-related factors in driving health outcomes [1]. Progress on this front, however, should not be assumed or taken for granted, given the normative pressures and practical constraints facing police officers who must navigate tensions between historically punitive models of law enforcement and the more recent push toward embracing a harm reduction approach.

## Conclusion

The current study set out to assess the psychometric properties of an adapted scale: the Group-Based Law Enforcement Mistrust Scale (GBLEMS). This scale showed strong reliability and validity and is well suited as a multi-item scale to capture race-based law enforcement mistrust. As hypothesized, the GBLEMS was associated with being Black or African American and other race-based experiences of discrimination and race-based medical mistrust. There were also positive associations between the GBLEMS and *direct* experiences with law enforcement (e.g., having been arrested because of one’s race) and one measure of vicarious experiences with law enforcement (e.g., having a recent sexual partner who had been incarcerated). In sum, considering law enforcement mistrust as a component of the risk environment may have important implications for individual health and public health.

## Data Availability

The datasets generated and analyzed during the current study are not publicly available due to the fact that the consent form our participants signed stated that, “No one but the study staff and investigators will have access to your study records.”

## Abbreviations

GBMMS: Group-based medical mistrust scale
GBLEMS: Group-based law enforcement mistrust scale
PWIDs: People who inject drugs
US: United States
